# Supplementary material for: Association between over-indebtedness and antidepressant use: A cross-sectional analysis
Source: PLoS One. 2020 Jul 24;15(7):e0236393. doi: 10.1371/journal.pone.0236393 (PMC7380887; doi:10.1371/journal.pone.0236393)
Supplement: Attachment — Submitted filename: Response_to_reviewers.pdf [file pone.0236393.s001.pdf]

Response to reviewers

PONE-D-19-32005
Association between Over-indebtedness and Antidepressant Use: A Cross-sectional Analysis
PLOS ONE

Journal Requirements

When submitting your revision, we need you to address these additional requirements.

1. Please ensure that your manuscript meets PLOS ONE's style requirements, including those for file naming. The PLOS ONE style templates can be found at http://www.journals.plos.org/plosone/s/file?id=wjVg/PLOSOne_formatting_sample_main_body.pdf and http://www.journals.plos.org/plosone/s/file?id=ba62/PLOSOne_formatting_sample_title_authors_affiliations.pdf

Response: We appreciate your remark and updated the manuscript to meet PLOS ONE’s style requirements.

2. We note that you have indicated that data from this study are available upon request. PLOS only allows data to be available upon request if there are legal or ethical restrictions on sharing data publicly. For information on unacceptable data access restrictions, please see http://journals.plos.org/plosone/s/data-availability#loc-unacceptable-data-access-restrictions.

In your revised cover letter, please address the following prompts:

a) If there are ethical or legal restrictions on sharing a de-identified data set, please explain them in detail (e.g., data contain potentially identifying or sensitive patient information) and who has imposed them (e.g., an ethics committee). Please also provide contact information for a data access committee, ethics committee, or other institutional body to which data requests may be sent.

b) If there are no restrictions, please upload the minimal anonymized data set necessary to replicate your study findings as either Supporting Information files or to a stable, public repository and provide us with the relevant URLs, DOIs, or accession numbers. Please see http://www.bmj.com/content/340/bmj.c181.long for guidelines on how to de-identify and prepare clinical data for publication. For a list of acceptable repositories, please see http://journals.plos.org/plosone/s/data-availability#loc-recommended-repositories.

We will update your Data Availability statement on your behalf to reflect the information you provide.

Response: We understand your request and explained the restrictions on sharing data from this study in our revised cover letter in more detail.

> “The data on over-indebted participants (OID survey) are not publicly available as these contain potentially identifying participant information that could compromise participants’ privacy. Therefore, data requests may be sent to the ethics committee of the Medical Faculty of the University of Bonn (Email: ethik@uni-bonn.de).

Data from the general population survey (DEGS1) conducted by the Robert Koch Institute is available to use by the scientific community on application as Public Use Files. Data can be requested upon application from the Research Data Centre: https://www.rki.de/EN/Content/Health_Monitoring/Public_Use_Files/application/application_node.html.“

Review Comments to the Author

Reviewer #1

The article stablishes a correlation between over-indebtedness and antidepressant use using cross-sectional analysis.
Over-indebtedness is inferred by presence in the OID survey which comprises clients of debt advice agencies. (independent variable)
Antidepressant use is seen as a proxy for mental-health disorders like depression/anxiety but also sleeping disorders and chronic pain. (dependent variable)

Comments:

Antidepressant use can both be interpreted as presence of mental disorder and access to health-care.
When being in debt compromises access to health-care or medication, association between mental health need and actual use of medication can be compromised.
In lines 229 to 233 the authors give a brief explanation about how health-care and medication is provided in Germany. The text should make it clearer that being in debt doesn’t get in the way of access to health care and medication. Otherwise those who are worst off wouldn’t report antidepressant use and the inference would be compromised.

“In a cross-sectional survey among clients of debt advice agencies in Germany 65.2 percent of participants had not filled a prescription due to cost in the past year [39]. In Germany, co-payments of 5 to 10 euros are required for each prescribed medication for adults covered by statutory health insurance [40]. Thus, the actual need for antidepressants might be underestimated in the present study.”

It mentions that being in debt does compromise complience to treatment in most cases (65.2%). The author should explain how this doesn’t compromise the premise the reporting antidepressant use in the survey is linked to mental health suffering. Not only the actual need for antidepressant use can be underestimated but it could also go the opposite direction.

Response: We agree with the reviewer that it might be useful to clarify access to health care and medication in Germany specifically for over-indebted individuals. Over-indebted individuals in Germany do have access to health services when enrolled in statutory health insurance, however co-payments apply for prescription medication as well as hospital care, medical aids, rehabilitation and home nursing. Therefore, over-indebted patients may be bound to weigh competing financial commitments and spending on necessities, such as (various) medications owing to cost.

As patterns of cost-related medication nonadherence are complex, we recently examined this specific aspect in detail to estimate the prevalence and to assess factors associated with cost-

related medication nonadherence among over-indebted individuals in Germany[1]. Patients that are enrolled in statutory health insurance can apply for reimbursement or a waiver of co-payments if these exceed a 2 % ceiling of gross household income per annum (1% for the chronically ill). Nevertheless, the prevalence of CRN among participants of the OID survey was 33.6%. The chronically ill were found to have significantly greater odds of CRN than those without a chronic illness (aOR 1.96; 95% CI 1.27–3.03).

We also found that over-indebtedness significantly increased the odds of pain (aOR 1.30; 95%-CI 1.07–1.59) after adjusting for socioeconomic, demographic and health factors[2]. However, the over-indebted were significantly less likely to use pain medication compared to the general population after adjustment (aOR 0.76; 95%-CI 0.58–0.99).

Overall, these findings indicate that over-indebted individuals can access medications but might selectively cut back on medication use due to co-payments. This is in line with the few available studies that have examined variations in medication underuse by disease and medication types[3]. Thus, on the one hand, we assume that over-indebted individuals frequently use antidepressants due to mental health suffering. Several studies have shown increased odds of mental illness such as psychoses [7] and depression [11–17] across diverse over-indebted populations. Those affected possibly choose to spend limited financial resources on co-payments for antidepressants in favour of other medications such as pain medication. On the other hand, we assume that we underestimate the prevalence of mental illness as not all patients use medication, specifically antidepressants, in part, owing to cost. The chronically ill may be particularly susceptible to cost-related medication nonadherence when facing high needs for medication. Thus, the present explorative study provides only a rough estimate of mental illness underlying antidepressant use among the over-indebted in Germany.

We have revised the Discussion section to briefly describe these aspects as follows:

> "On the other hand, previous studies indicate that individuals with poor mental health are significantly more likely to report cost-related medication nonadherence (CRN) such as not filling prescriptions, skipping doses or splitting pills [36]. Likewise, over-indebtedness has been found to be associated with CRN [37,38,13]. In a nationally representative study of Americans older than 50 years, 32.1 percent of those considered over-indebted reported CRN [13]. Among over-indebted individuals that participated in the present study, the prevalence of CRN was 33.6% [1]. The chronically ill were found to have significantly greater odds of CRN than those without a chronic illness. In Germany, co-payments of 5 to 10 euros are required for each prescribed medication for adults covered by statutory health insurance [40]. Thus, over-indebted patients may be bound to weigh competing financial commitments and spending on necessities, such as (various) medications owing to co-payments. In line with these findings, this explorative

[1]Warth J, Puth M-T, Tillmann J, Beckmann N, Porz J, Zier U, et al. (2019) Cost-related medication nonadherence among over-indebted individuals enrolled in statutory health insurance in Germany: a cross-sectional population study. BMC Health Serv Res. 19: 887.

[2]Warth, J; Puth, M-T; Tillmann, J; Porz, J; Zier, U; Weckbecker, K; Münster, E (2019) Over-indebtedness and its association with pain and pain medication use. Preventive medicine reports 16: 100987.

[3]Such as Piette JD, Heisler M, Wagner TH (2004) Cost-related medication underuse among chronically ill adults: the treatments people forgo, how often, and who is at risk. Am J Public Health. 2004. 94(10):1782-7. or Piette, JD, Heisler M, Horne R, Alexander G (2006) A conceptually based approach to understanding chronically ill patients' responses to medication cost pressures. Soc Sci Med 62 (4): 846–857.

study provides only a rough estimate of mental illness underlying antidepressant use among the over-indebted in Germany, and might underestimate the association between over-indebtedness and antidepressant use." *Discussion section (page 12f., lines 227-246)*

"Moreover, it cannot be excluded that the over-indebted who neither seek debt advice nor medical services are underrepresented in the OID survey. This, in turn, might overestimate antidepressant use in this population whereas CRN might have the opposite effect." *Discussion section (page 14, lines 270-273)*

As our findings of cost-related medication nonadherence in the over-indebted sample have been published elsewhere (see above), we summarized relevant key aspects to avoid replication. Please do not hesitate to contact us if you would like us to provide further details in the present manuscript.

Databases

The authors merge two separate databases. One from the OID Survey which comprises a sample of clients of debt advice agencies and assumes that the whole of the sample is over-indebted. The survey took place between July and October 2017 in North-Rhine Westphalia (NRW). (n=699)

The other database comes from a nation wise survey in Germany (German Health Interview and Examination Survey for Adults (DEGS1)) with data colected betweew November 2008 and 2011 (n=7115).

What should be clarified is if data used from the DEGS1 comprises the whole Germany or of just the NRW part of the sample. Using a NRW sample from the DEGS1 and comparing it with the OID sample, would reduce statistical power but make the samples more comparable.

Response: We appreciate the reviewer's careful revision and have revised the manuscript to clarify the points raised. First, we highlighted that DEGS1 comprises population data on a national level whereas the OID survey was carried out in the German federal state of North Rhine-Westphalia only. We assume that the over-indebted participants of the regional OID survey are comparable to the over-indebted population at national level. First, approved debt advisory centres offer similar counselling services throughout Germany. Second, the demographic structure of the federal state in terms of gender, age distribution and foreigners is similar to the national average[4].

In the Results section (page 8, lines 157-160) we describe the data sets as follows:

"The merged sample comprised 7814 participants, of which 699 were over-indebted clients of debt advice agencies in the German federal state of North Rhine-Westphalia (OID survey), and 7115 adults living in Germany that participated in the national health survey DEGS1."

---

[4]Statistische Ämter des Bundes und der Länder (2014) Zensus 2011. Bevölkerung nach Geschlecht, Alter, Staatsangehörigkeit, Familienstand und Religionszugehörigkeit. Available: https://www.statistikportal.de/sites/default/files/2017-06/zensus_2011_bevoelkerung.pdf. Accessed 25 April 2020.

The authors should also clarify if they can be sure there's no overlapping of the databases since they the data was collected independently, (i.e. – The same person being interviewed in bith surveys). And if there can be overlap how this does not interfere with the results.

Response: We thank the reviewer for this constructive comment. We assume that the databases merged in the present study overlap, if at all, to a slight extent. Data collection for the general population sample was carried out between 2008 and 2011 whereas the over-indebted sample was recruited in 2017. Moreover, given the serious financial problems of the over-indebted participants, we consider those affected a 'hard-to-reach' population that is likely to be underrepresented in the general population survey as the latter requires participants to complete interviews, physical examinations, tests and laboratory analyses. Nevertheless, we cannot exclude that the DEGS1 sample also includes over-indebted individuals. We pointed this out in the Discussion section as follows (page 14, lines 263-269):

> "Furthermore, a broadly accepted definition of over-indebtedness is not yet available [1]. Therefore, we defined OID participants that were seeking debt advisory agencies as over-indebted and assumed DEGS1 participants not to be over-indebted due to lack of data on indebtedness. However, it is likely that the DEGS1 population also includes individuals that are over-indebted according to this definition. This procedure might have yielded attenuated estimates of the association between over-indebtedness status and antidepressant use."

Reviewer #2

This study examined the association between over-indebtedness and antidepressant use. Considering previous studies have reported the relationship between over-indebtedness and mental well-being, the authors should address and what the current study adds to what is already known in the Introduction

Response: We appreciate the reviewer's careful review of our manuscript and constructive comments. The present study is a first explorative study conducted in Germany that aims to examine patterns of mental illness and associated medication use among over-indebted individuals – given the national regulations on over-indebtedness as well as the cultural and social context. As the latter circumstances are assumed to influence not only the legal but also psychosocial consequences such as mental illness linked to over-indebtedness, the present study allows to compare previous findings that are mainly based on US data with explorative data of a German over-indebted population.

According to the reviewer's suggestion, we specified what the present study adds to existing research in the Introduction section (page 3f., lines 43-69):

> "Research on social determinants of health has a long tradition but most studies have focused on socioeconomic status (SES) measures including education, income and occupation [5]. However, individuals across the socioeconomic spectrum may face over-indebtedness which cannot be considered interchangeable with the standard socioeconomic measures [2,5]. Several studies have shown that measures of over-indebtedness are associated with various health outcomes, independent of standard SES measures [6]. Besides physical morbidity, e.g. diabetes [7], obesity [8] and pain [9,10], over-indebtedness has consistently been found to be associated with mental illness, including psychoses [7] and depression [11–17].

Prescriptions of antidepressants continue to increase worldwide [18,19]. While depressive disorders are the main indication for antidepressant prescriptions, antidepressants are also prescribed for other indications such as anxiety, sleeping disorders and pain [20,21]. Thus, antidepressant use may not only serve as an indicator of mental illness but also provide a first insight into access to mental health care. Depressive disorders are currently one of the three leading causes of disability globally and the associated disease burden continues to increase [22,23]. A large body of evidence suggests a social gradient in the prevalence of depression: Those in low socioeconomic positions are more frequently affected by depression [24–26]. Some studies have suggested an association between SES measures and antidepressant use but evidence is inconsistent [27–30]. Previous studies mainly examined US data. However, there are variations in legal as well as social consequences of over-indebtedness across countries that may affect the prevalence of mental illness and medication use considerably. Therefore, the aim of the present explorative study was to investigate the association between over-indebtedness and antidepressant use in Germany."

The study examined the harmful impact of over-indebtedness, but it is not clear why the authors used antidepressant use as the outcome measure. Antidepressant use is better measure for mental well-being? Antidepressant use is related to both presence of mental illness and access to mental health care. Over-indebtedness may affect negatively mental wellbeing but reduce access to medicine. The impact of over-indebtedness on mental well-being could be underestimated. Otherwise, did this study aim to examine access to medicine in people with mental illness due to over-indebtedness? Then, mental illness should be adjusted for in the statistical model. The analytical model depends on research question, however, which is not clear. Further, it the implications of the finding is not clear.

Response: We thank the reviewer for pointing out that these aspects need further clarification.

a. We are aware of the fact that the measure of antidepressant use cannot provide an exact estimate of the prevalence of mental illness because not all of those affected will use medication, specifically antidepressants, for various reasons including help-seeking or cost. Nevertheless, we considered antidepressant use a relevant measure to explore the prevalence of common mental disorders in the over-indebted population for the following reasons: Firstly, given that antidepressants are mostly available only on prescription, the advantage of this measure is that it specifically takes underlying mental illnesses into account that have been diagnosed by a clinician. Secondly, previous studies have suggested that mental illness such as depression is widely spread in various over-indebted populations [11–17]. However, our knowledge of both the prevalence of mental illness in the over-indebted in Germany, and the association between over-indebtedness and antidepressant use that are mainly prescribed for depressive disorders, is limited. Finally, data on antidepressant use was available for both the over-indebted sample (OID survey) and the general population sample (DEGS1) which allowed the present analysis. In the context of existing research, this explorative study therefore aimed to examine antidepressant use in the over-indebted in Germany for the first time.

   We have revised the Introduction section (page 3f., lines 43-69) and Discussion section (page 14f., lines 274-284) to clarify the focus on antidepressant use of the present explorative study and to highlight limitations:

   "Research on social determinants of health has a long tradition but most studies have focused on socioeconomic status (SES) measures including education, income and

occupation [5]. However, individuals across the socioeconomic spectrum may face over-indebtedness which cannot be considered interchangeable with the standard socioeconomic measures [2,5]. Several studies have shown that measures of over-indebtedness are associated with various health outcomes, independent of standard SES measures [6]. Besides physical morbidity, e.g. diabetes [7], obesity [8] and pain [9,10], over-indebtedness has consistently been found to be associated with mental illness, including psychoses [7] and depression [11–17].

Prescriptions of antidepressants continue to increase worldwide [18,19]. While depressive disorders are the main indication for antidepressant prescriptions, antidepressants are also prescribed for other indications such as anxiety, sleeping disorders and pain [20,21]. Thus, antidepressant use may not only serve as an indicator of mental illness but also provide a first insight into access to mental health care. Depressive disorders are currently one of the three leading causes of disability globally and the associated disease burden continues to increase [22,23]. A large body of evidence suggests a social gradient in the prevalence of depression: Those in low socioeconomic positions are more frequently affected by depression [24–26]. Some studies have suggested an association between SES measures and antidepressant use but evidence is inconsistent [27–30]. Previous studies mainly examined US data. However, there are variations in legal as well as social consequences of over-indebtedness across countries that may affect the prevalence of mental illness and medication use considerably. Therefore, the aim of the present explorative study was to investigate the association between over-indebtedness and antidepressant use in Germany." *Introduction section (page 3f., lines 43-69)*

"Given that antidepressants are mostly available only on prescription, the advantage of this measure is that it specifically takes underlying mental illnesses into account that have been diagnosed by a clinician. However, it is not possible to distinguish indications for antidepressant use or identify unmet need based on available data. Finally, the processes of collection of data on medication use differed between the two survey populations. In contrast to DEGS1, OID relied solely on self-reporting which might have led to minor under-reporting. However, several studies suggest high validity of self-reported use of various medication types [43], such as antidepressants [44,45]. Despite limitations, this study provides first evidence of an association between over-indebtedness and antidepressant use and enhances the scientific understanding of socioeconomic inequalities in mental health." *Discussion section (page 14f., lines 274-284)*

b. We agree with the reviewer that our results are likely to underestimate the association between over-indebtedness and antidepressant use as over-indebtedness may both have detrimental effects on mental health and reduce access to medication. We have modified the Discussion section to emphasize this aspect (page 12f., lines 227-246):

"On the other hand, previous studies indicate that individuals with poor mental health are significantly more likely to report cost-related medication nonadherence (CRN) such as not filling prescriptions, skipping doses or splitting pills [36]. Likewise, over-indebtedness has been found to be associated with CRN [37,38,13]. In a nationally representative study of Americans older than 50 years, 32.1 percent of those considered over-indebted reported CRN [13]. Among over-indebted individuals that participated in the present study, the prevalence of CRN was 33.6% [41]. The chronically ill were found to have significantly greater odds of CRN than those without a chronic illness. In Germany, co-payments of 5 to 10 euros are required for each prescribed medication for

adults covered by statutory health insurance [40]. Thus, over-indebted patients may be bound to weigh competing financial commitments and spending on necessities, such as (various) medications owing to co-payments. In line with these findings, this explorative study provides only a rough estimate of mental illness underlying antidepressant use among the over-indebted in Germany, and might underestimate the association between over-indebtedness and antidepressant use."

c. The primary objective of the present study was to provide a first insight into patterns of antidepressant use as an indicator of mental illness in the vulnerable population of the over-indebted in Germany. The findings suggest that over-indebted patients in Germany appear to have a high demand for antidepressants, and have access to antidepressants as they are more likely to use antidepressants than the general population.
In our statistical model we adjusted for chronic disease which include mental disorders to account for the effect of such long-term health conditions. Due to the limitations of the available data described in the Discussion section (page 14, lines 259-284), we cannot answer the question with certainty, to what extent those who need medication for mental illness can access treatment. Given that previous studies have shown that the over-indebted frequently suffer from mental illnesses, we would like to emphasize that barriers to access to medications for over-indebted patients may nevertheless affect our findings. Further research is necessary to quantify to what extent over-indebted patients with mental disorders are unable to access antidepressants and to identify mechanisms that explain this relationship.

   As shown above, we have, accordingly, revised the Introduction section to clarify the primary objective of this explorative study (page 3f., lines 43-69) and revised the limitations of our study in the Discussion section (page 14f., lines 274-284) to emphasize that we cannot draw conclusions on unmet need for medication based on the available data but "only provide only a rough estimate of mental illness underlying antidepressant use among the over-indebted in Germany […]".

Over-indebtedness was defined with OID survey, information from clients of debt advice agencies. All people in over-indebtedness are included in the OID survey? I wonder people who seek more help are more likely to be clients of the agencies and to be prescribed with antidepressant. It should be described whether and how using OID survey to define over-indebtedness has advantages.

Response: We appreciate the reviewer's suggestion to describe the recruitment of over-indebted individuals in more detail. By recruiting clients of debt advice agencies for the OID survey, we were able to collect such comprehensive data on over-indebted individuals' medication use in Germany for the first time. Although this population group can be considered a 'hard-to-reach' group owing to serious financial problems, we achieved a high response rate of 50.2%. Therefore, we assume that this study takes a broad range of over-indebted individuals into account. Nevertheless, like other population studies, the OID survey does not include all over-indebted individuals. On the one hand, those who seek help from debt advice agencies might be more likely to use health care as consultations are free of charge. On the other hand, clients of debt advice agencies might be more likely to underuse medication due to co-payments when taking into account that it is severe financial problems that will prompt those affected to seek debt advice (see page 12f., lines 227-246). Therefore, we acknowledge that antidepressant use in the over-indebted included in the study is possibly overestimated or underestimated. Despite these limitations, this explorative study provides first evidence of patterns of antidepressant use in the over-indebted which, however, requires further research.

In order to emphasize these limitations, we have modified the Discussion section as follows (page 14, lines 259-273):

> “A few limitations need to be acknowledged when interpreting the findings of this study. Due to the cross-sectional nature of the present study, the causal direction of the observed association remains unclear. It is both possible that mental illness underlying antidepressant use results from over-indebtedness and mental illness contributes to over-indebtedness by reducing the capability to manage debt [11,6,7]. Furthermore, a broadly accepted definition of over-indebtedness is not yet available [1]. Therefore, we defined OID participants that were seeking debt advisory agencies as over-indebted and assumed DEGS1 participants not to be over-indebted due to lack of data on indebtedness. However, it is likely that the DEGS1 population also includes individuals that are over-indebted according to this definition. This procedure might have yielded attenuated estimates of the association between over-indebtedness status and antidepressant use. Moreover, it cannot be excluded that the over-indebted who neither seek debt advice nor medical services are underrepresented in the OID survey. This, in turn, might overestimate antidepressant use in this population whereas CRN might have the opposite effect.”
